# Rapid Fabrication of Cell-Laden Alginate Hydrogel 3D Structures by Micro Dip-Coating

**DOI:** 10.3389/fbioe.2017.00013

**Published:** 2017-02-24

**Authors:** Atabak Ghanizadeh Tabriz, Christopher G. Mills, John J. Mullins, Jamie A. Davies, Wenmiao Shu

**Affiliations:** ^1^School of Engineering and Physical Sciences, Heriot-Watt University, Edinburgh, UK; ^2^Centre for Integrative Physiology, University of Edinburgh, Edinburgh, UK; ^3^Centre for Cardiovascular Science, University of Edinburgh, Edinburgh, UK; ^4^Centre for Synthetic Biology, University of Edinburgh, Edinburgh, UK; ^5^Department of Biomedical Engineering, University of Strathclyde, Glasgow, UK

**Keywords:** alginate, hydrogel, biofabrication, micro dip-coating, cell-laden, vascular structures

## Abstract

Development of a simple, straightforward 3D fabrication method to culture cells in 3D, without relying on any complex fabrication methods, remains a challenge. In this paper, we describe a new technique that allows fabrication of scalable 3D cell-laden hydrogel structures easily, without complex machinery: the technique can be done using only apparatus already available in a typical cell biology laboratory. The fabrication method involves micro dip-coating of cell-laden hydrogels covering the surface of a metal bar, into the cross-linking reagents calcium chloride or barium chloride to form hollow tubular structures. This method can be used to form single layers with thickness ranging from 126 to 220 µm or multilayered tubular structures. This fabrication method uses alginate hydrogel as the primary biomaterial and a secondary biomaterial can be added depending on the desired application. We demonstrate the feasibility of this method, with survival rate over 75% immediately after fabrication and normal responsiveness of cells within these tubular structures using mouse dermal embryonic fibroblast cells and human embryonic kidney 293 cells containing a tetracycline-responsive, red fluorescent protein (tHEK cells).

## Introduction

Conventional 2D, monolayer cell culture still remains the main approach for the study of cell biology, regenerative medicine, and drug discovery (Gies et al., 2002). However, the relevance of 2D cell cultures, compared to 3D physiological conditions is limited and 3D cell culture approaches are important in narrowing the gap between *in vitro* and *in vivo* studies (Birgersdotter et al., 2005; Bellis et al., 2013; Edmondson et al., 2014; Zhao et al., 2014; Imamura et al., 2015). Current methods for the fabrication of 3D scaffold materials include self-assembly (Peck et al., 2011), solvent casting (Thadavirul et al., 2014), dry freezing (Offeddu et al., 2015), and electrospinning (Bini et al., 2004; Chu et al., 2014). However, the main barrier in conventional scaffold-based tissue engineering approaches is the inability to position living cells precisely to mimic 3D tissue. Repopulation of decellularized tissues and organs has been reported to regenerate 3D tissue and can be used as a platform for drug discovery and organ transplantation, but this approach relies on the availability of donated organs so cannot be scaled up indefinitely (L’Heureux et al., 1998; Crapo et al., 2011; Wilson et al., 2013).

3D biofabrication (Yamaguchi et al., 2012; Faulkner-Jones et al., 2013, 2015; Kang et al., 2013, 2016; Gudapati et al., 2014; Ghanizadeh Tabriz et al., 2015; Hochleitner et al., 2015; Li et al., 2015; Ouyang et al., 2015a; Groll et al., 2016) is a very promising emerging field that gives experimenters the ability to position cell-laden bio-inks precisely into a predesigned 3D structure. Recent studies on 3D bioprinted tumor models using HeLa cells (Zhao et al., 2014) show the cells to be more chemoresistant than in normal 2D monolayer culture, making the 3D system a potentially better model for study of real cancer cells from patient biopsies.

There is, however, a significant problem with current 3D biofabrication approaches: although they can generate simple and precise 3D structures, they rely on specialized bioprinting machinery that is not easily accessible to many cell biologists. Therefore, a simple, controllable, and straightforward 3D biofabrication method that does not involve complicated machinery such as bioprinting platforms would be very valuable and effective to create biomimetic 3D structures.

In this study, we present a new, inexpensive, and simple approach to rapid biofabrication that generates cell-laden tubular structures with tuneable micron resolution and the ability to generate multiple layer hydrogel tubular structures. This approach could potentially be suitable in fabricating microvasculature *in vitro* and other tubular shaped structures within the human body.

Alginate hydrogel was used as the main substrate for this study as it is currently the most widely used biomaterial for 3D biofabrication due to its ease of use, biocompatibility, and the control that can be exerted over its biological half-life (Mørch et al., 2006; Ghanizadeh Tabriz et al., 2015). To validate this new 3D biofabrication technique, mouse dermal fibroblast cell viability was monitored within the tubular structures over 6 days. Furthermore, tetracycline-inducible gene expression in a human cell line was used to demonstrate that cells within the fabricated tubular structures were alive and responsive to external signals.

## Materials and Methods

### Cell Culture

All cell lines used in this study were cultured in 5.0% CO_2_ at 37.0°C. Human embryonic kidney 293 cells containing a tetracycline-responsive red fluorescence protein (tHEK cells) were kindly donated by Dr. Elise Cachat (Centre for Integrative Physiology, Edinburgh University, UK) and cultured in Dulbecco’s Modified Eagle Medium (Sigma D5796) with 5% fetal bovine serum (Biosera, FB-1090/500). Mouse dermal embryonic fibroblasts, kindly donated by Mrs. Audrey Peter (College of Medicine & Veterinary Medicine, Edinburgh University, UK), were cultured in Minimum Essential Medium (Sigma M5650) supplemented with 5% fetal bovine serum and 1% l-glutamine (Invitrogen, 25030-081).

### Materials and Regents

In this study, sodium alginate solutions of 6 and 8% (w/v) (Product number: W201502, sodium alginate, Sigma-Aldrich, Gillingham, UK) prepared in deionized water and collagen type I 0.4% (w/v) from rat tail and dissolved in 20 mM acetic acid (Product number: C3867, Sigma-Aldrich, Gillingham, UK) were used as scaffold. A total of 100 mM CaCl_2_ (calcium chloride dihydrate CAS number: 010035-04-8, purity ≥ 99%, Sigma-Aldrich, Gillingham, UK) and 55 mM BaCl_2_ (barium chloride dihydrate, CAS number 10326-27-9, ≥99.999% trace metals basis, Sigma-Aldrich, Gillingham, UK) prepared in deionized water were used as the cross-linking reagents. Metal bars that were used to carry cell experiments were purchased from OK International (TE718150PK, OK International) where the metal part was extracted from the needles.

### Cell-Laden Hydrogel Preparation

Sodium alginate 8% (w/v) was sterilized by gamma radiation (IBL-637 CIS-BioInternational gamma irradiator, France) with 10 Gy at the rate of 1 Gy/min. For experiments including cells, 0.5 mL of a cell suspension of either tHEK cells or mouse dermal embryonic fibroblast cells, at a concentration of 8 × 10^6^ cells/mL, was mixed with 1 mL of 8% (w/v) sodium alginate solution to result a 6% (w/v) sodium alginate solution with cell concentration of 2.67 × 10^6^/mL, respectively. Similarly, 0.4% (w/v) collagen solution was prepared containing 8% (w/v) sodium alginate and sterilized by Gamma radiation with 10 Gy at the rate of 1 Gy/min. A total of 0.5 mL of each cell type with concentration of 8 × 10^6^/mL were separately added to result a 0.26% (w/v) collagen and 6% (w/v) sodium alginate solution at a cell concentration of 2.67 × 10^6^/mL.

### Alginate Hydrogel Single-Layer Tubular Structure Fabrication by Micro Dip-Coating

**Figure 1:**
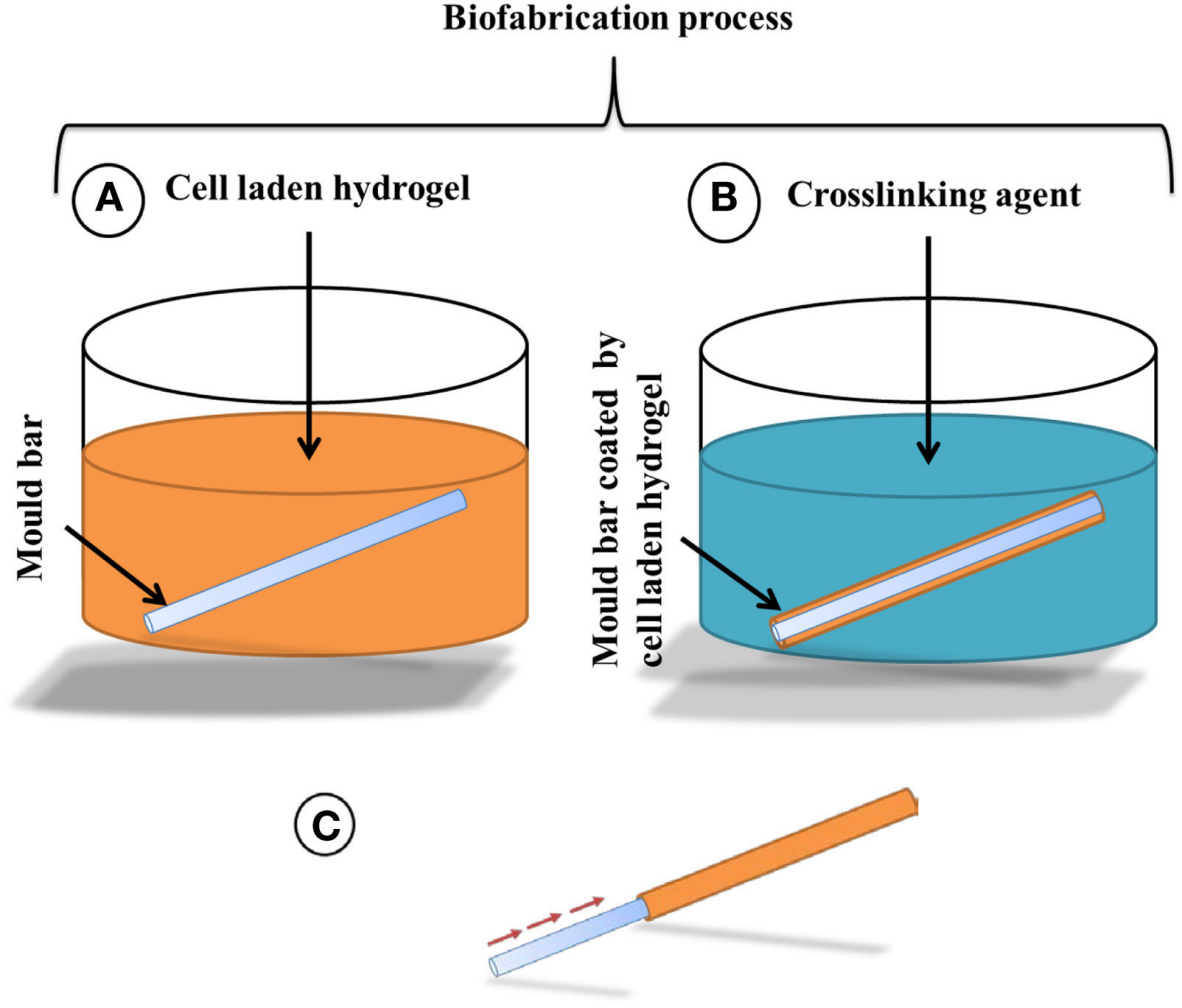
**Schematic drawing of the alginate hydrogel tubular structure fabrication process, (A) a metal bar mold is dipped into 6% (w/v) sodium alginate to coat the surface by a thin layer of cell-laden alginate hydrogel, followed by (B) the exposure to 55 mM BaCl_2_ or 100 mM CaCl_2_ to fully cross-link the sodium alginate layer for 2 min and then (C) the cross-linked alginate layer will be pulled out from the mold as a hollow tube**.

**Figure 2:**
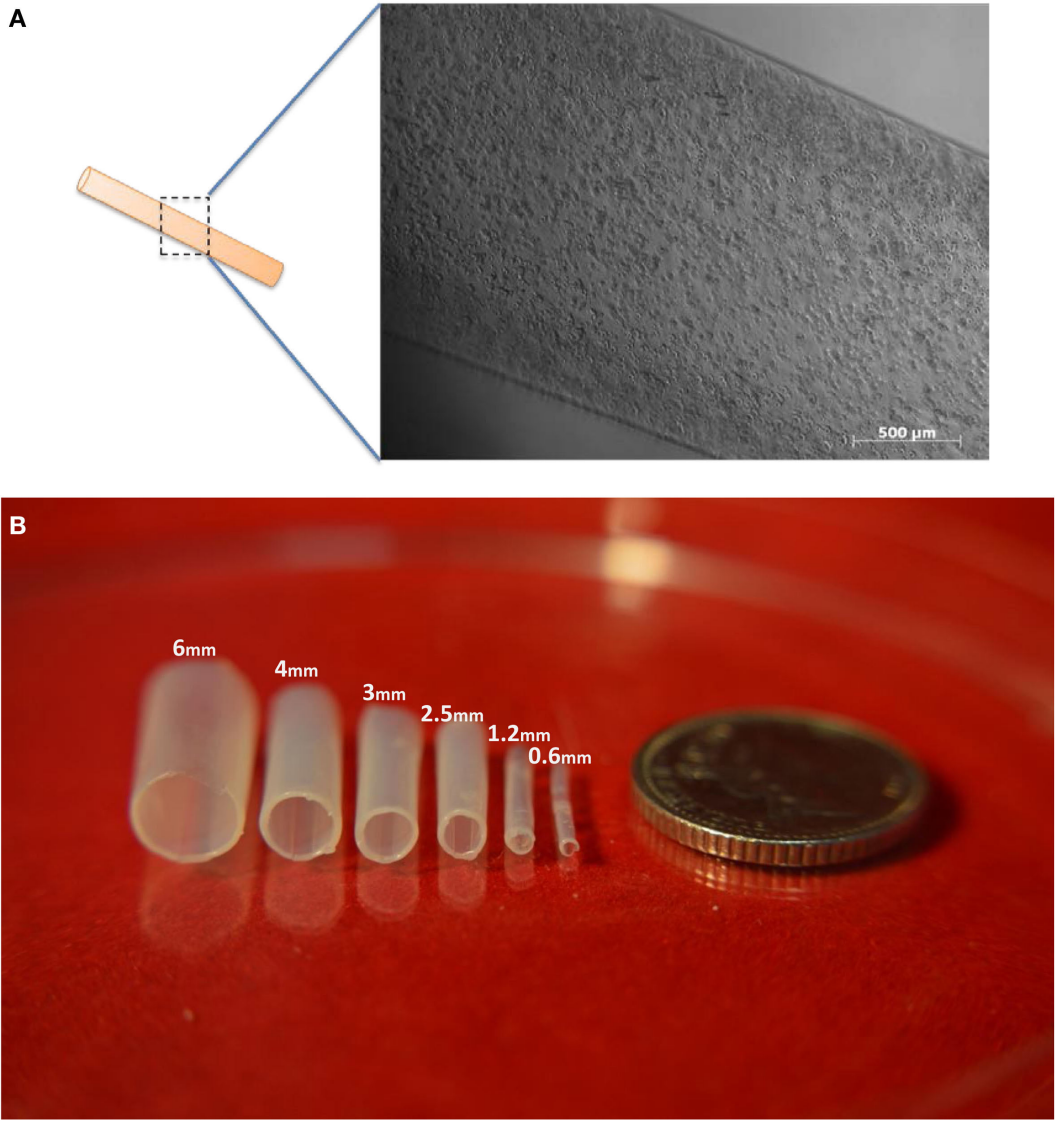
**Images of tubes made from alginate**. **(A)** A cell-laden tubular alginate hydrogel structure fabricated via dip-coating. **(B)** Alginate hydrogel tubular structures fabricated by the dip-coating method. Fabricated single-layer alginate hydrogel tubular structures with various diameters with descending diameters from left to right. Scale bar: 5p.

Stainless steel metal bars were chosen, because of their wettability: bars with a range of diameters (down to 600 µm) were used as the mold for tubular structure fabrication. The metal bars were kept in ethanol overnight, prior to use, to sterilize them. As depicted in Figure 1A, the mold was first dipped into a 6% (w/v) sodium alginate solution, with or without cells, or dipped into collagen-sodium alginate solution, with or without cells, for about 3 s and then removed. A thin layer of the cell-laden hydrogel was left coated on the surface of the metal bar. The metal bar was then dipped into filter-sterilized cross-linking reagents (100 mM CaCl_2_ or 55 mM BaCl_2_) for 2 min to cross-link the sodium alginate of the coated layer shown in (Figure 1B). The cross-linked cell-laden alginate hydrogel was then gently pulled out by tweezers from one end of the metal bar mold, leaving a hollow tubular structure of cross-linked cell-laden hydrogel shown in Figure 1C and Figure 2A. Depending on the diameters of the metal bar mold, hollow tubular structures with different inner diameters down to 600 µm have been fabricated. For biological studies, cell-laden tubular hydrogel structures were fabricated using a metal bar with 1.2 mm diameter.

### Alginate Hydrogel Multiple Layer Tubular Structure Fabrication

To fabricate multilayered tubular structures, the mold was dipped into sodium alginate for 3 s and then dipped in to the cross-linking reagent (100 mM CaCl_2_ or 55 mM BaCl_2_) for 2 min to cross-link and form the first layer of the tubular structure. The remaining residual solution was removed carefully by absorption by sterilized tissue paper. Then, the cross-linked layer, still on the mold, was dipped into to the sodium alginate again for 3 s to be coated by the second layer of sodium alginate and then dipped into the cross-linking agent (100 mM CaCl_2_ or 55 mM BaCl_2_) for 2 min to cross-link and form the second layer. This procedure was repeated as many times as necessary to reach the number of layers desired within the tubular structures.

### Flow Experiment through Cross-Linked Alginate Hydrogel Tubular Structures

The flow experiment was designed to investigate whether the fabricated alginate hydrogel tubular structures are sealed against liquid flow within the tubular structure. An alginate hydrogel tubular structure made using a 4 mm rod was fabricated followed by the dip-coating protocol. A total of 60 mL of water with a red dye was prepared and loaded into a 60 mL syringe. The syringe was then loaded into a syringe pump, and the tubular structure was fitted to the syringe tip. The syringe pump flowrate was set to 20 mL/s for 3 min.

### Viability Assay

Tubular structures containing mouse embryonic dermal fibroblasts were fabricated using 1.2 mm diameter rods according to the above protocol and cultured for 6 days. A LIVE/DEAD^®^ Cell Vitality Assay Kit (L34951, Life Technologies) was used according to the manufacturer’s protocol. Briefly, the medium was replaced with fresh medium containing 500 nM c_12_-resazurin and 10 nM SYTOX Green before incubating at 37°C, 5% CO_2_ for 15 min.

### 3D Cell Imaging and Viability Testing of Mouse Embryonic Dermal Fibroblast Cells

Confocal images of mouse embryonic dermal fibroblast cells were taken on a Nikon A1R FLIM confocal microscope. Z-stacks (range of 176 µM, 5 µM steps) of each sample were captured using a Nikon A1R FLIM confocal microscope. Images were analyzed using Imaris software to investigate the viability of the cells encapsulated within the tube walls fabricated by 1.2 mm mold at day 1, day 3, and day 6 of culture.

### Inducing the tHEK Cells with Tetracycline and Imaging

Tubular structures containing tHEK cells within the tube walls were fabricated using 1.2 mm rods by the fabrication protocol and incubated for 24 h. After 24 h of incubation, the medium was replaced with fresh medium contacting tetracycline at concentration of 1 µg/mL and then incubated for further 48 h. Images of the induced tHEK cells were captured after 72 h using Zeiss Axiovert Immunofluorescence microscope.

## Results and Discussion

### Biofabrication of Alginate Hydrogel Tubular Structures with Variable Diameters and Alginate Concentrations

Our strategy for producing cell-laden tubular structures was to dip a stainless steel rod into a suspension of cells in an alginate-based buffer and then to transfer the rod, still coated with a thin layer of this suspension, into a solution of divalent cations that would cross-link the alginate into a hydrogel (Figure 1A). A variation of the strategy was to repeat this process, building up thicker and thicker hydrogels on the rod, before the rod and hydrogels were separated to leave a cell-laden, hydrogel tube.

We began by testing the idea using acellular solutions of alginate. Tubular structures were successfully fabricated using 6% (w/v) alginate and stainless steel rods of inner diameters ranging from 0.6, 1.2, 2.5, 3, 4 to 6 mm (Figure 2B), using either 100 mM CaCl_2_ or 55 mM BaCl_2_ solution to promote the cross-linking procedure. The thickness of the tube walls formed on rods of 0.6, 1.2, 2.5, 3, 4, and 6 mm were 126 ± 6, 143 ± 5, 171 ± 9, 181.6 ± 13, 206.6 ± 6, and 220 ± 7 µm, respectively (the ± figures indicating SD). These results indicated an interesting correlation between the tube wall thickness and the inner diameter of the tube, where a metal rod (mold) with greater diameter used during fabrication resulted in a larger tube thickness.

**Figure 3:**
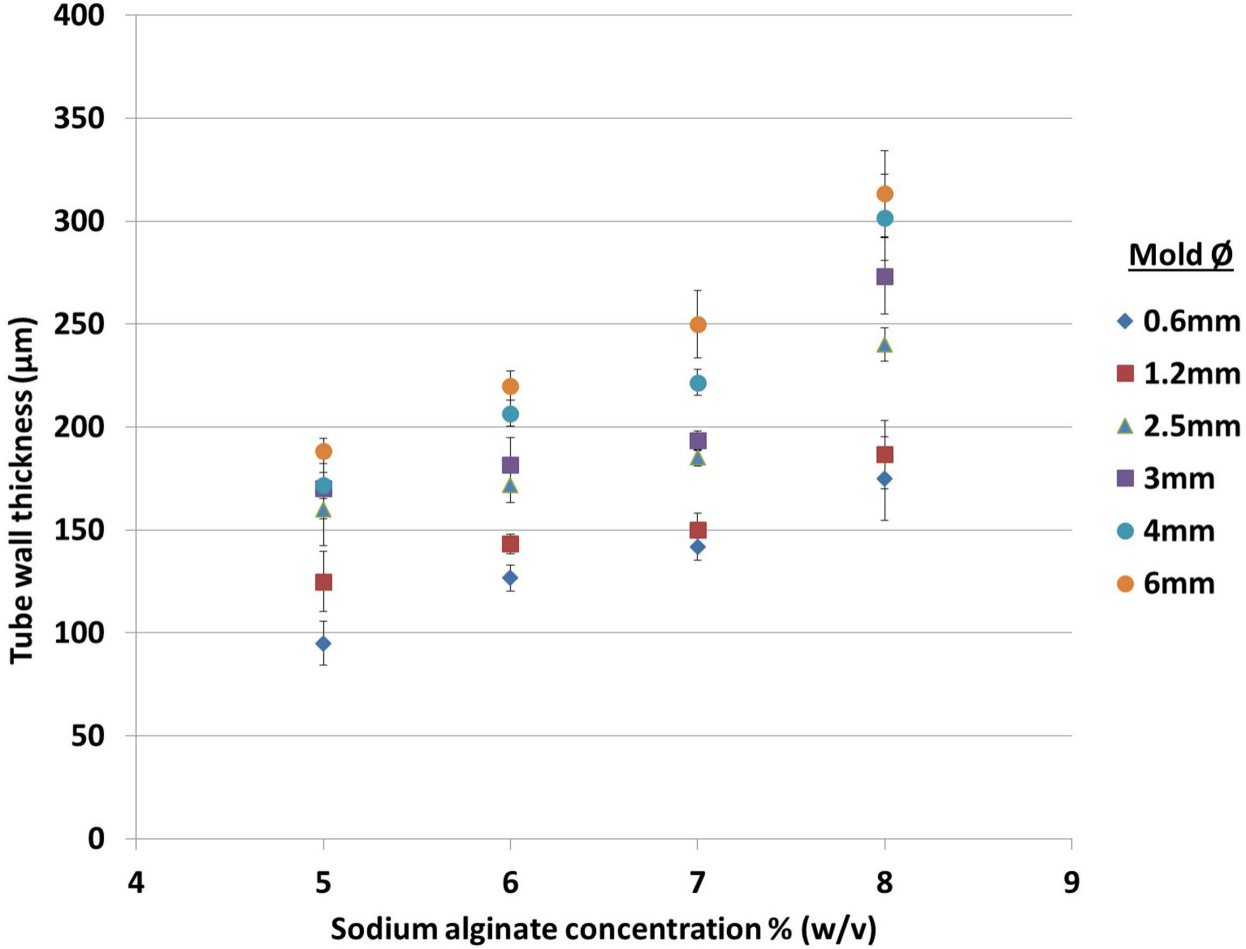
**Effect of sodium alginate concentration and rod diameter on the wall thickness of tubes fabricated using one dip in the alginate solution**. The rod diameters are indicated to the right of the graph.

To characterize further the relationship between rod diameter, alginate concentration, and resulting wall thickness, solutions of 5, 6, 7, and 8% (w/v) alginate were used to fabricate tubular structures. The measurements (Figure 3) indicated that wall thickness increased approximately linearly with respect to sodium alginate concentration. 5% (w/v) alginate was the minimum concentration capable of fabricating tubular structures in this system: tubes made with lower concentrations had insufficient rigidity to maintain the integrity of the structure when the rod was removed. It should be noted that this minimum is probably specific to the sodium alginate used in this study and that other sodium alginates with different viscosities and molecular weights may require different alginate concentrations and different concentrations of cross-linking reagents to fabricate the tubular structures, which may or may not result in a tubular structure with either smaller or bigger wall thickness.

**Figure 4:**
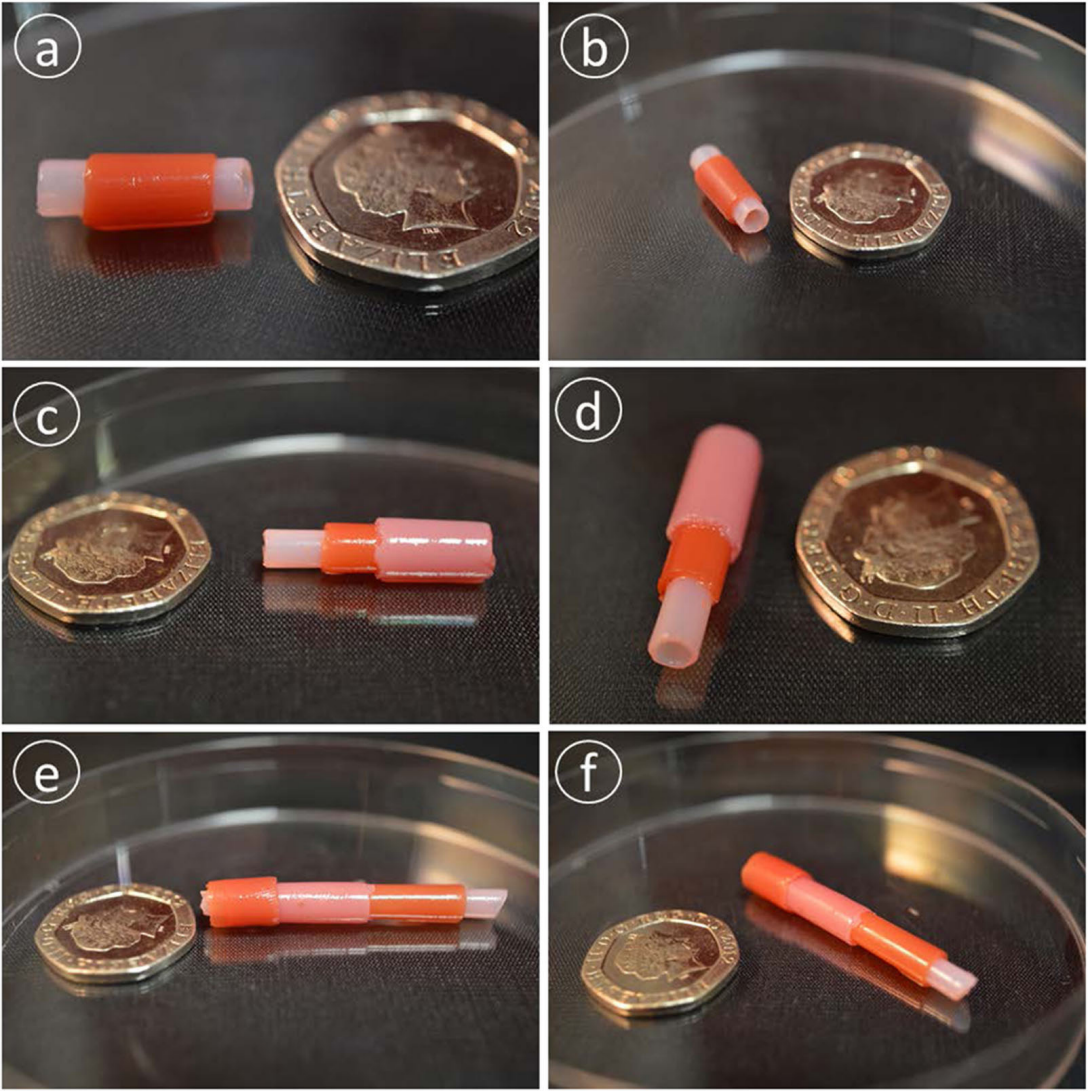
**Multilayer alginate structures**. Panels **(A,B)** show two layers, **(C,D)** three layers, and **(E,F)** four layers. Each layer was distinguished by making it with an alginate solution with different dyes. Scale bar: 20p coin.

The idea of repeating the dip-then-cross-link cycle to build and create thicker walls was tested by transferring cross-linked hydrogel, still on the rod, back into the alginate solution, and cross-linking again. This procedure, done one to three times, successfully fabricated thicker walls. The distinct layers are visible in transverse sections of the tubes (Figure 4). Potentially, this approach could position different cell types within each layer with the ability to fine-tune each layer to the desired thickness depending upon the application. However, the tube wall thickness after the micro-coating is dependent on the wettability of the coating surface. Therefore, the wall thickness of the secondary layer would depend on the wettability of the first coated layer and hence the thickness of the second layer may vary with different batches of material from the data presented here.

**Figure 5:**
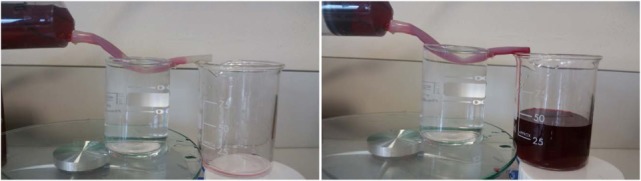
**Flow experiment through a single-layer alginate hydrogel tubular structure**. The red dye is pumped form a syringe, through the fabricated tube to the beaker on the right. The dye is transferred and does not leak either into water (the beaker on the left) or air (note the absence of dye on the table).

In addition as shown in Figure 5, the tubular structure is completely liquid tight from the start to the end of the flow and provided a safe and sealed environment for liquid flow through the tubular structure. The fabricated cell-laden tubular structures here with tuneable diameters could be used as a disease model to mimic and study the flow behavior and flow–cell interactions inside blood vessels.

### Cell Viability in Alginate and Collagen-Alginate Hydrogels

**Figure 6:**
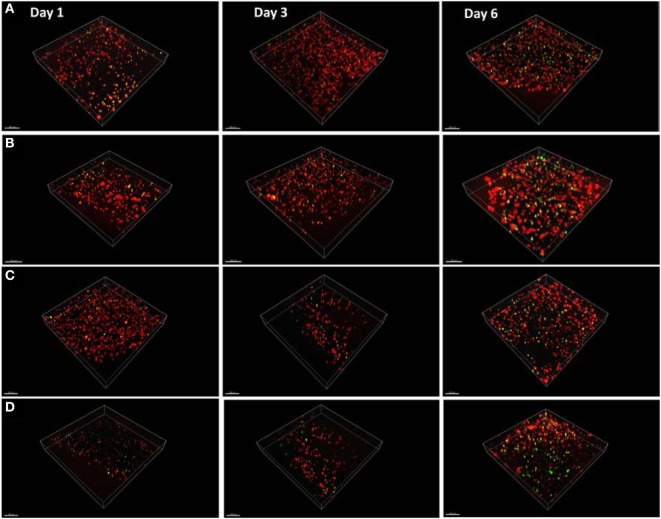
**Mouse fibroblast cell lines cultured within the tube walls of (A) alginate hydrogel cross-linked by calcium, (B) alginate hydrogel cross-linked by barium, (C) alginate-collagen cross-linked by calcium, and (D) alginate-collagen cross-linked barium over 6 days**. Red stain indicates live cells and green stain indicates dead cells. Scale bar: 100 µm.

**Figure 7:**
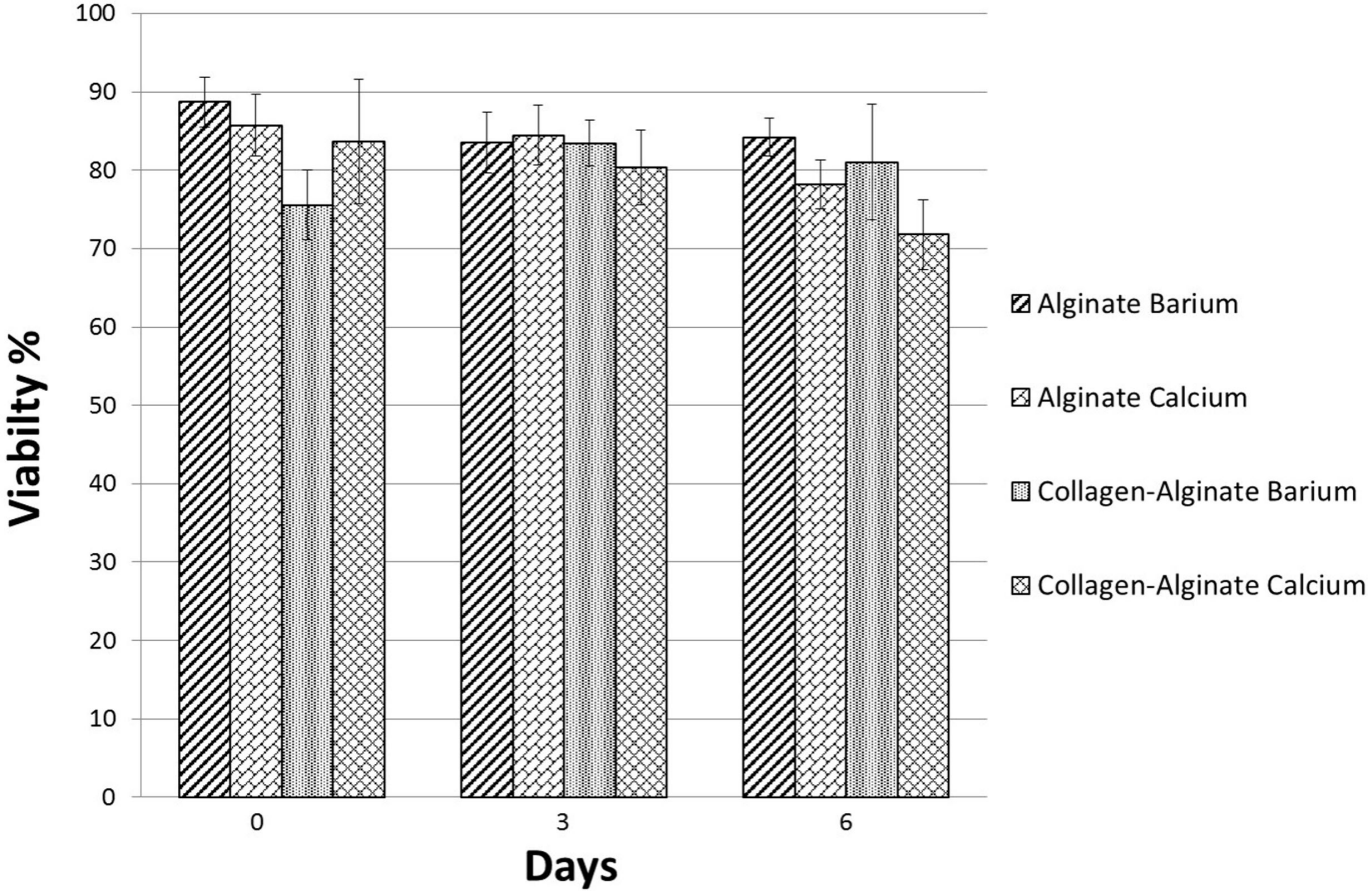
**Viability of mouse embryonic dermal fibroblast cells within the tube walls over 6 days of culture fabricated using alginate and collagen-alginate cross-linked by 100 mM CaCl_2_ and 55 mM BaCl_2_**.

Having demonstrated the basic tube fabrication technique using acellular alginate, we went on to produce cell-laden tubes. Tubular structures containing mouse dermal fibroblast cells were fabricated using the dip-coating method described above, but with NIH-3T3 mouse fibroblasts suspended in the alginate solution before the rod was dipped in it. In some experiments, collagen I was added to the alginate. Two different cross-linking solutions were tried: BaCl_2_ and CaCl_2_. Cells were stained using a LIVE/DEAD^®^ Cell Vitality Assay, which stains living cells red and dead cells green and allowed viability to be monitored through 6 days of culture for each condition. In all conditions, cells could clearly be detected within the tube walls (Figure 6). All conditions showed good cell viability. Cells within alginate hydrogel tubes and collagen-alginate cross-linked with 55 mM BaCl_2_ had the highest viability of 84 ± 2.4% and 81 ± 7.4%. Cell viability in alginate hydrogel, and alginate-collagen, cross-linked by CaCl_2_ was 78 ± 3.1 and 72 ± 4.4% (Figure 7). The results showed a good viability after 6 days of culture within the tubular structures for all conditions indicating good biocompatibility of the gel. However, a slight decrease in viability in collagen-alginate mixture cross-linked by 100 mM CaCl_2_ and 55 mM BaCl_2_ was observed and was probably due to lower pH level of the collagen solution creating a harsher environment for the cells.

### Cell Proliferation

**Figure 8:**
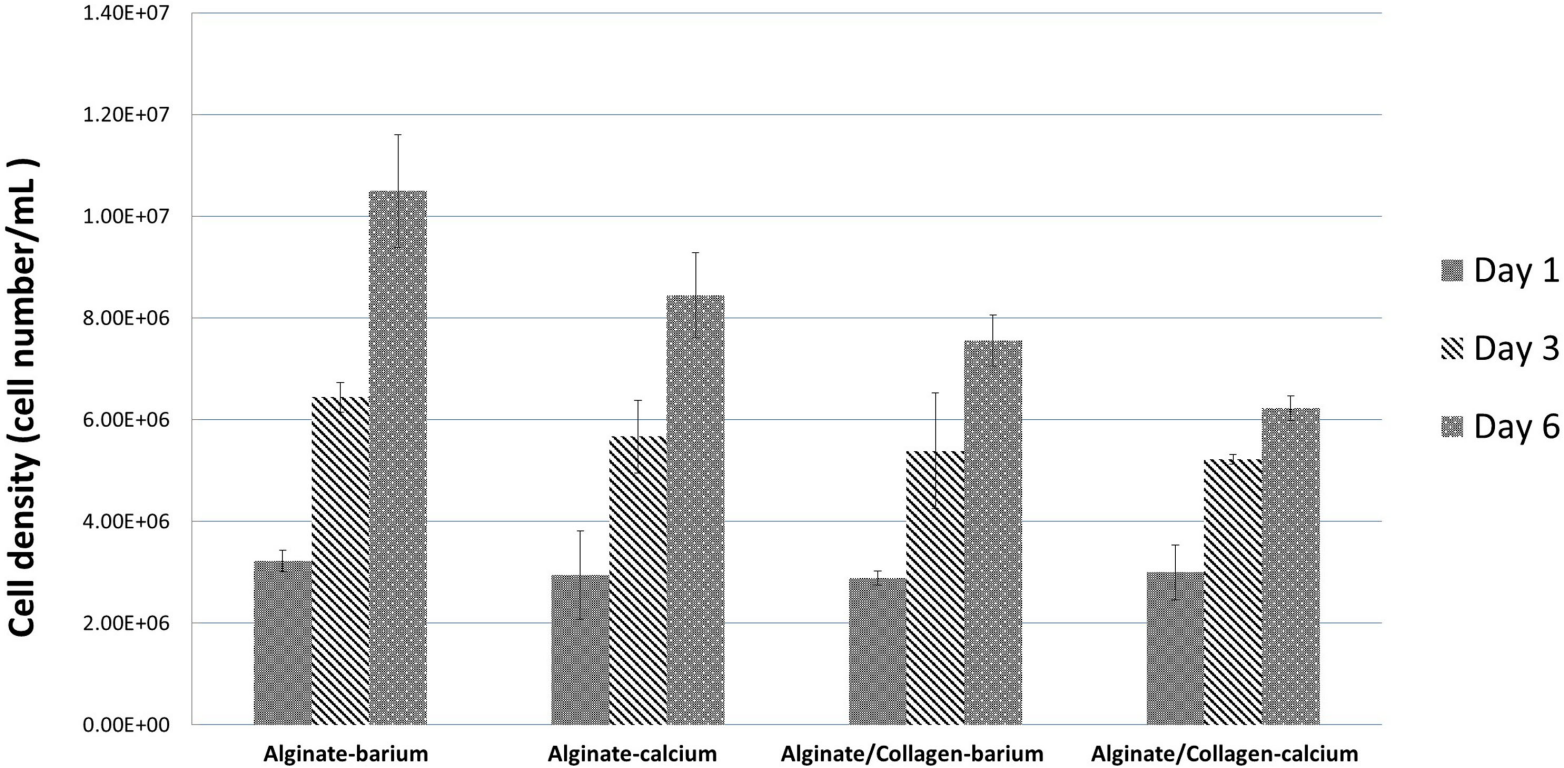
**Fibroblast cell numbers within the tubular structures fabricated by four different conditions at day 1, day 3, and day 6 of culture**.

Confocal images of the mouse dermal fibroblast cells were taken for the four different conditions of fabrication at day 1, day 3, and day 6 of culture as shown in Figure 6. Three random areas within each tubular structure was selected at 300 µm × 300 µm × 50 µm to count the cell numbers through Imaris software. Based on the results shown in Figure 8 for all four conditions, cells have grown and proliferated in some parts of the gel and have formed small clusters within the fabricated tube walls. The following results indicated that the fabricated gel has a good permeability and porosity allowing nutrition and oxygen to penetrate into the gel as well as allowing the waste to be extracted from the gel. These optimum conditions created a suitable enough extracellular matrix for cells to grow while supporting cell to cell interaction in some parts of the gel. Figure 6 summarizes the cell density within the tubular structures fabricated in four different conditions throughout 6 days in culture. Cell density is considerably higher in alginate cross-linked with BaCl_2_ where initially also cell viability results indicated that highest viability was achieved in alginate cross-linked with BaCl_2._

### Responsiveness of Cells to Small Molecules

**Figure 9:**
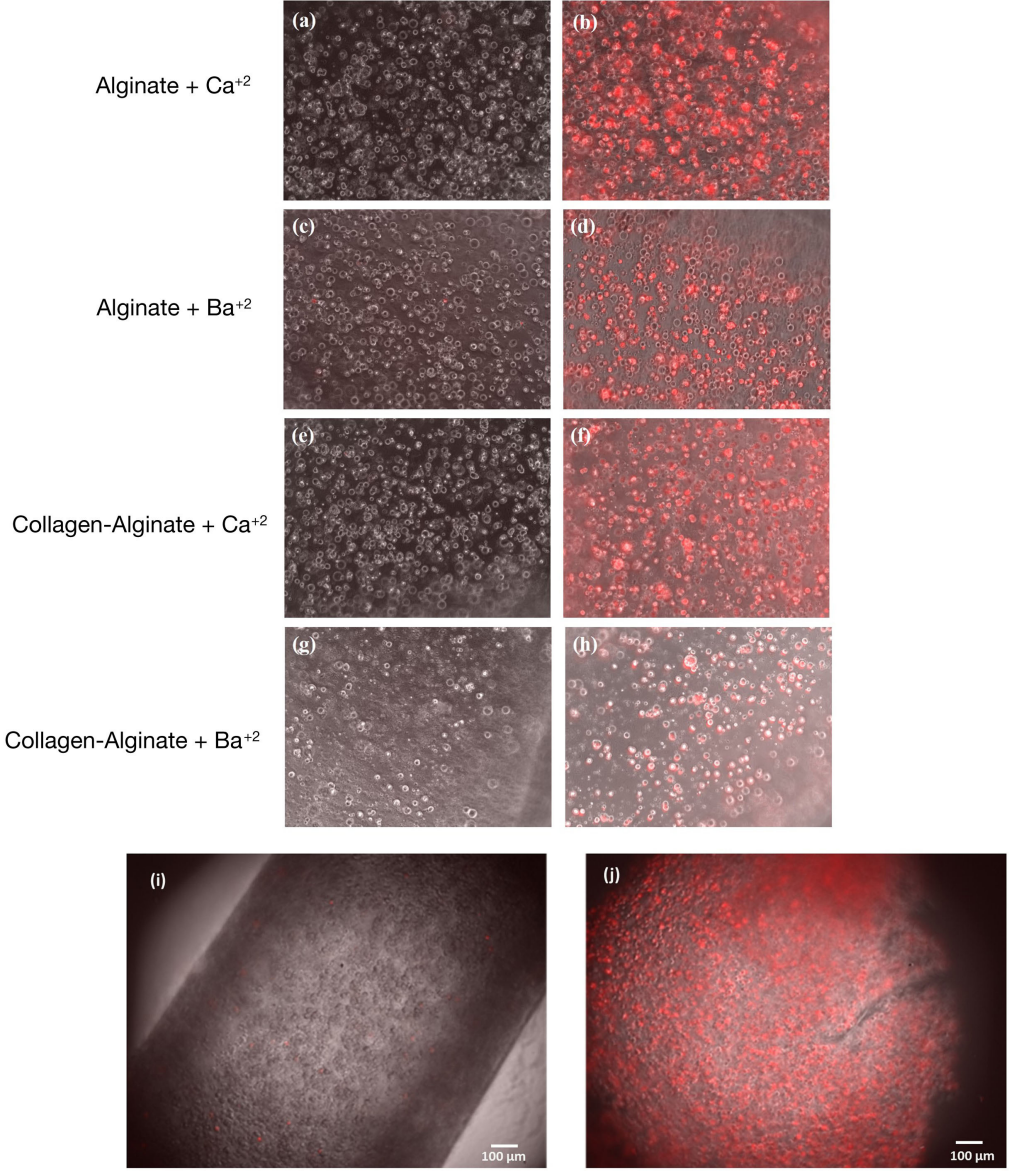
**Responsiveness of cells in the wall to small signaling molecules**. Panels **(A,C,E,G)** show tHEK cells cultured in tubes made from, and cross-linked by, the reagents indicated, but not exposed to tetracycline. Panels **(B,D,F,H)** show tHEK cells in tubes made the same way, but then exposed to tetracycline after 24 h and cultured for a further 48 h. Red fluorescent protein is robustly induced. Panels **(I,J)** show low-magnification images of the tubes containing cells before and after induction with tetracycline.

To validate whether the walls of the tube allow cells to access small signaling molecules, and whether they show normal gene expression responses to these, we used a tHEK cell line that activates expression of red fluorescent protein (RFP) in response to tetracycline (Wong et al., 2007; Ouyang et al., 2015b; Cachat et al., 2016). The tHEK cells were cultured for 24 h within the fabricated tube walls, again made using the four alternative methods of tube manufacture (with and without collagen, and using either CaCl_2_ or BaCl_2_ to cross-link). They were then cultured for a further 48 h in the presence or absence of tetracycline. Cells cultured with no tetracycline could be detected but showed no red fluorescence (Figures 9A,C,E,G,I). Cells exposed to tetracycline showed a robust induction of RFP expression in all four conditions (Figures 9B,D,F,H,J).

## Conclusion

In this study, we have developed a new rapid 3D biofabrication technique for making alginate hydrogel tubular structures. Importantly, this technique can be done in a standard biology lab and requires no cell sheet technologies (Sekine et al., 2006; Kubo et al., 2007; Matsuda et al., 2007; Othman et al., 2015) or other complex tubular fabrication approaches that require expensive and complicated machinery systems (Wang et al., 2005; Takei et al., 2006; Johnson et al., 2010; Hume et al., 2011; Yuan et al., 2012; Onoe et al., 2013; Sakai et al., 2013; Sher et al., 2015). Tubular structures from submillimeter, or a few hundred micron range, to greater diameters with an ability to control the thickness of the tube walls can be fabricated using the micro dip-coating technique. This approach might incorporate other biomaterials within alginate hydrogel to help cell biologists to bypass lengthy, complicated and expensive fabrication approaches. They can then use the dip-coating method to fabricate 3D tubular structures with living cells in their preferred extracellular matrix. The fabrication method is gentle to live cells while maintaining high cell viability over 6 days within the tubular structures cross-linked by CaCl_2_ and BaCl_2_. It also leaves cells free to interact with small molecules such as the tetracycline used as a demonstration here. This method presents a promising potential for fabricating tubular structures that are better models of anatomy than 2D cultures are, and may be suitable for a range of tubular tissues including embryonic kidney, lymph vessels, blood vessels, trachea, and intestine.

## Author Contributions

AGT, JM, JD, and WS designed the experiments; AGT and CM performed the experiments; AGT, CM, JM, JD, and WS wrote the paper.

## Conflict of Interest Statement

The authors declare that the research was conducted in the absence of any commercial or financial relationships that could be construed as a potential conflict of interest.
